# “Just as expensive as sending him to college:” barriers and perceptions of treatment in justice-involved youth

**DOI:** 10.1186/s40352-024-00289-2

**Published:** 2024-07-23

**Authors:** Corey McBrayer, Annie Turner, Mackenzie Whitener, Zachary W. Adams, Leslie Hulvershorn, Tamika C. B. Zapolski, Matthew C. Aalsma

**Affiliations:** 1 Adolescent Medicine, PennState Health, 905 Governor Rd Ste 200, Hershey, PA 17033 USA; 2 Marion University College of Osteopathic Medicine, 3200 Cold Spring Rd, Indianapolis, IN 46222 USA; 3 Section of Adolescent Medicine, Department of Pediatrics, Indiana University School of Medicine, 410 W 10th St, Indianapolis, IN 46202 USA; 4 Department of Psychiatry, Indiana University School of Medicine, 410 W 10th St, Indianapolis, IN 46202 USA

**Keywords:** Qualitative research, Adolescent health, Substance use disorder, Criminal justice

## Abstract

**Background:**

Justice-involved youth have higher rates of substance use disorders (SUDs) than the general population. Many do not connect with or complete treatment, leading to recidivism. This qualitative study explores perceptions and barriers to treatment in this population.

**Results:**

Justice-involved youth participating in a larger study focused on access to SUD treatment were interviewed about available treatment and justice system involvement. Twenty-one dyads (youth and a guardian) and 3 individual guardians (total *N* = 45) were interviewed by phone. Inclusion criteria were youth aged 14–17 involved in the justice system that screened positive for SUD. Youth sample was 43% male. Thematic analysis guided the process. The study was Indiana University Institutional Review Board approved (#1802346939).

Data was interpreted within the ecological system theory. Youth barriers included willingness to engage in treatment, time constraints/scheduling conflicts, and low perceived usefulness of treatment. Major guardian themes included high cost of treatment, lack of communication by the justice system about treatment, youth unwillingness or disinterest to engage in treatment, and limited program availability.

**Conclusions:**

The barriers to treatment for justice-involved youth are multifaceted and occur across the spectrum of levels of the ecological system, which include parents, peers, social systems, and cultural elements. Many youth and guardians suggested improvements for their interactions with the juvenile justice system. Further examination is needed of current policy implementation to address these concerns.

## Background

Substance use is a major health concern for adolescents (Garofoli, 2020; Miller et al., 2021). Adolescent substance use is related to increases in the risk of mental health disorders (Kolp et al., 2018), impaired social functioning (Hicks et al., 2010), poor school performance (Andrade, 2014), risky sexual behavior (Shorey et al., 2015), and development of substance use disorders (SUDs) during adolescence and adulthood (Stone et al., 2012; Winters & Lee, 2008). Justice involved youth (JIY) are at especially high-risk for substance use with up to 75% of JIY meeting diagnostic criteria for SUD (Harzke et al., 2012; McClelland et al., 2004; Teplin et al., 2002; Wasserman et al., 2005) as compared with 4.5% in community counterparts (Quality, 2019). JIY are also more likely to experience mental illness with estimates of 50–70% of JIY meeting criteria for at least one mental health disorder (Peters et al., 2017; Yonek et al., 2019) and 10–14% of JIY meeting criteria for both SUD and a mental health disorder (Abram et al., 2003; Teplin et al., 2005). Untreated mental health and substance use disorders increase their risk of suicidality, criminal activity, and recidivism (Gordon et al., 2004; Kolp et al., 2018; Schubert et al., 2011; Yonek et al., 2019). With effective treatment, many of these consequences may be avoided (Cuellar et al., 2004). However, studies have shown that the majority of JIY do not engage in treatment. It is estimated that 50–80% (Elkington et al., 2020) of JIY diagnosed with an SUD are unable to access necessary substance use services while in the justice system and even fewer access services after release (< 16%) (Yonek et al., 2019).

Barriers to treatment for JIY are multi-faceted and can arise from the youth themselves, the people/agencies they interact with, and the systems and society that surround them. The ecological systems theory provides a useful framework to conceptualize the influences of multiple systems on young people (Kerwin et al., 2015). Ecological theory states that a child’s environment is composed of five interrelated systems: the microsystem containing the child’s immediate environment such as family, teachers, and peers, the mesosystem consisting of interactions between microsystems, the exosystem containing social structures that indirectly impact the child, the macrosystem consisting of cultural elements, and the chronosystem which encompasses changes over a lifetime. Interactions occur bidirectionally between these systems and the child, and both the child and the different systems influence each other (Bronfenbrenner, 1974). Previous qualitative and quantitative studies have identified barriers at many of these levels including caregiver engagement, caregiver and youth denial (i.e., do not perceive a need for treatment), distrust of the medical system, overburden on the family at the microsystem and mesosystem levels, such as cost for services, insurance coverage, and transportation, lack of training at the exosystem level, and stigma and lack of prioritization at the macrosystem level (Belenko et al., 2017; Yonek et al., 2019).

Although qualitative studies have investigated JIY access to care generally, none have specifically focused on accessing substance use services. Additionally, it is important that studies stay up to date on current barriers faced by this population, as this will likely improve their care. Our study explored the lived experiences of JIY and their guardians, which is important for policy development and improvement within the juvenile justice system when caring for these youth. We sought to deepen our understanding of challenges faced by JIY and their guardians as they seek care.

## Methods

The current project was a part of a broader implementation effort to identify JIY with substance use treatment needs and engage them in evidence-based substance use treatment. Below, we briefly describe the methods for this study. Additional information can be found in the published study protocol (Aalsma et al., 2019). The university’s institutional review board approved the study (July 28, 2018). All participants gave informed consent/assent for participation.

Interviews were conducted virtually via telephone over an 18 month period with participants recruited from 2 semi-rural counties in a midwestern state in the USA. Interviews were conducted by trained female or non-binary research staff at an academic health center with either a Masters or Doctoral degree. Interviews were recorded, transcribed via a HIPAA compliant company, and de-identified by research staff. Interviewers had no prior relationships with research participants. Table 1 includes examples of questions included in the interview guide, which was semi-structured. Participants qualified to take part in the interview based on their participation in the parent study, having scored positive on an SUD screening tool, and both parent/guardian and youth agreeing to participate. Participants were compensated $25 for completion of an interview. All participants were only interviewed once. Those who declined interviews cited time constraints or disinterest in participation as reasons for declining.

**Table 1:** Example interview guide questions

Sample guardian questions	Sample youth questions
Tell me a bit about yourself and your child.	Tell me a bit about yourself and what you do for fun.
What has your experience with the JJ system been like?	Think back to when you were first arrested or completed an intake with a probation officer. Were you placed on probation?
Have you experienced challenges with staff?	What do you remember being communicated about treatment? About substance use treatment?
What could be done to make your experience better?	Did you hear anything from court staff once you got home [if attended court]?
What do you remember being communicated about treatment?	Where you interested in treatment? Assess perspective on need for treatment
If your child went to court, what did you hear from court officials about treatment?	Where the treatments available appropriate? Why or why not?
Was your child interested in treatment through our program?	Have you accessed substance use or mental health treatment outside our program? If so, how was it?
What made it difficult to participate in treatment?	Was there any treatment that you were interested in that was not available?
What made one treatment option more appealing than another?	What would have made it easier to participate in treatment?

A thematic analysis approach was used for coding the interviews (Terry et al., 2017). After deidentification, transcripts were read and coded by trained masters or doctoral level researchers using Atlas.ti. Each transcript was reviewed by at least two researchers for inter-coder agreement. Categories and themes were identified by researchers as they emerged and were discussed collectively to identify connections between categories. New themes and models were compared to the transcript data until saturation was reached. Interviews were continued until thematic saturation was reached. Each coder reviewed all data once the interviews concluded and discussed each theme and transcript until agreement was reached.

## Results

For this study, a total of 21 youths and 24 guardians were interviewed. The mean age of the youths was 15.1 years (SD = 1.0), with 43% (*n* = 9) of participants identifying as male. Of the guardians interviewed, the mean age was 40.0 years old (SD = 7.0), with 13% (*n* = 4) of guardians identifying as male (see Table 2 for additional demographic information). All interviews took no longer than one hour with a median interview time of approximately 20 min each. For the thematic analysis, the most common barrier themes are discussed below divided by youth and guardian, along with suggestions from both for system improvement. See Tables 3, 4 and 5 for additional quotes.

**Table 2:** Demographic data

Youth (*n* = 21)	Mean age (SD)	15.7 (1)
	Grade	8th = 2 (10%) 9th = 2 (10%) 10th = 6 (29%) 11th = 6 (29%) 12th = 3 (14%) Working on GED = 2 (10%)
	Gender	Male = 9 (43%) Female = 11 (52%) Other = 1 (5%)
	Race/Ethnicity	White = 11 (52%) African American = 4 (19%) Hispanic = 2 (10%) More than one race = 3 (14%) Other = 1 (5%)
Guardian (*n* = 24)	Mean age (SD)	40.0 (7)
	Gender	Female = 21 (87%) Male = 3 (13%)
	Race/ Ethnicity	White = 14 (58%) Black/African American = 6 (25%) Hispanic = 3 (13%) More than one race = 1 (4%)

**Table 3:** Treatment barriers identified by youth

**Youth interest**	“Really I just feel like it [treatment] doesn’t help because they just try to educate it…I feel like you can only change for yourself and not somebody else.” “the more I kept going to interviews [treatment sessions] I kind of lost interest in it because it felt like it was more being forced on me." “For me, to be honest, I don’t know what can help me because I don’t like talking about my problems. I don’t really want to solve them. I just want to lock them up and leave them over here. Like, basically, just get rid of them.” “No, not really [not interested in treatment], because no matter what anybody’s telling, I’m going to do what I want to do at the end of the day. My head will tell me about training technique, this is not going to help, this is what will help in real life. If I try to think about something, -read a book and color, but in real life, ain’t nobody likely to tell me, “no. Wrong move. Read a book. It was just her telling me to think like that and I can’t just think like that” “I just thought it was stupid. Other kids that do cocaine, heroin, speed, meth, and all that stuff. I’m just here smoking Mary Jane, ain’t nothing wrong with that. It’s legal in the States. Why can’t it be legal everywhere?”
**Negative JJ experience**	“But if anything, it [JJ experience] did push me to do it [use substances] more because of the stress of being arrested and the stress of all of these new issues that I’m facing and the worry of drug tests and everything else. It was a lot of stress on me at once, and I’m going to be bluntly honest with you. The day after I got arrested, I was like I’m just having consistent panic attacks. I’m like I need to smoke.” “They don’t help you at all. They basically just want you put in the system. They don’t really try to help you as much as you think they’re going to help.”
**Scheduling/time**	“I work a lot, so I don’t have really a lot of free time to do stuff like that [participate in treatment].” “I don’t go there [treatment] because I feel like I don’t have time. I have so much to do. I have a job. I go to school. I have all my friends. I don’t see how I would get that in there for real.”
**Type of treatment available**	“But like, everybody there in that class I had [previous treatment experience] was there for weed. Like a marijuana charge or whatever…me and all those people were there for the same thing, it’s kind of easier to talk about because they all understand where you’re coming from. But like, if you got a crack head in the corner, and you got someone that’s addicted to alcohol, and then someone who just smokes a blunt every now and then, I mean it’s a little difficult to understand their insight because we don’t really know what they’re going through or anything. So like, sometimes people don’t really want to talk about that to just anybody. It’s like, with those programs, they expect you to talk and I don’t just … I don’t know, like share extra. But sometimes people aren’t really comfortable with that.” “Less talking [would have made treatment participation easier]. I feel like I write more, like they need to figure out how people communicate.”
**Trust**	“I don’t talk about the things that I need to talk about, because that’s just not something I share with anybody. I share that with people I trust and I don’t trust very easily.” “I’d say the most difficult part was kind of gaining the trust for my therapist.”

**Table 4:** Treatment barriers identified by guardians

**Cost/insurance**	“It [treatment] was like just as expensive as sending him [youth] to college…Once they told me the price, I’m like, “Yeah, I cannot afford that. I couldn’t even afford that if I worked two jobs.””
**Youth interest**	“He [youth] said he really didn’t like it…he felt like it was just nonsense, like it was just a bunch of talking, and it didn’t help anything.”
**Lack of communication from JJ/CMHC**	“And then they [CMHC] were supposed to get back with us and set up further appointments and they have not, and I’ve made calls to them and I’m just told that they will reach out to us.” “I wake up in the morning, take him there [treatment facility] on Saturdays like I was instructed to. Then when I talked to the people there, nobody knew anything and no one had talked to anybody from juvenile probation. There was nothing in place.”
**No appropriate treatment available**	“Every single facility that we called, there were waiting lists. And when we would tell them what we were needing to be seen for, they were very hesitant to schedule her and would say, “Well, we’ll put you on the list and we’ll call you back.””

**Table 5:** Suggestions for improvement

**Less leniency from JJ**	“I [parent] actually think that they [JJ] were maybe too lenient on her, if she [youth] was to call every day for a drug test. That’s one of the things that she was supposed to do and then I would get the phone call saying that she needs to call in every day… there were sometimes that she did have her phone and he [probation officer] still contacted me, to have her keep on calling and she missed several days and really nothing was done.” “I [parent] think they should have been harder on him [youth] rather than just like, well, okay, don’t do this again.”
**More mental health awareness**	*Parent* “[JJ] should have some kind of program to where they help these children learn about their diagnoses and learn how to function with it. If they have real kids that are coming through the juvenile system with legit diagnoses. How come they don’t have a program that’s just like, listen, yes, you have extra challenges, but you need to learn how to live with them, how to cope with them. How to deal with them because once you enter society if you allow your diagnoses to take over your actions and behaviors, you are facing real consequences. Why don’t they have something like that?” *Parent* “Usually when you meet with a probation officer, it’s usually like, okay, did you pay your fees? Pee in this cup. How have you been? Are you working? If so, is it steady? Where do you live? Questions along those lines. From what I [parent] remember my experience because I’ve been on probation once. I don’t remember anybody ever asking me about my mental health. Or do I have any problems with my mental health, to where I may need some help? I don’t remember those conversations and I feel like maybe if somebody just asked are you okay? Have you been stressed about anything lately? Are you wanting to talk to anybody? Like here are the resources that are available to you, like that’s what I’m saying."
**Better communication**	*Parent* “I guess just better communication [would make it easier to participate in treatment]. You know what I mean? It’s like when he did the assessment, I don’t know what was going to be recommended or anything like that. So it’s like, I’m at a stand still trying to figure out what’s going on. What are they doing? You know what I mean?”
**Positive reinforcement**	“I [youth]feel like that’s a big step for me to stop and pass those drug tests, because I did it just because I didn’t want to be on probation or deal with them no more. But I feel like maybe if they would have, if they [JJ] give kids a little more recognition than they do I feel like some things wouldn’t go as bad as they do. Like, I know I shouldn’t want a reward for doing the right thing, but at the same time, sometimes people need that little extra push just to feel better about themselves and it would make them be like, okay, well they do recognize what I’m doing, maybe I should keep it up.”
**Decreased fees**	“At the end of it, I [parent] had to pay [the county] money but I just felt like I shouldn’t have paid for anything because truthfully, they didn’t do much of anything… It was like the administrative fee. I don’t know maybe to type in what happened. I had to pay somebody to do that. I had to pay the court fees, but we never actually went to court.” “I [parent] owe like $5,000…I don’t feel that I should be held accountable for that because he [youth] was old enough at that time. He was 17. I don’t think that I should be held accountable for his actions. I can’t control. I can see if he went out and he vandalized or damaged somebody’s property. That’s different. Him running away, then [JJ] locking him up, and I have to be held accountable. I don’t feel that that’s fair.”
**Improve continuity of care once probation is over**	*Parent* “So I feel like if they [courts] can offer them service during probation they should be able to offer them past that instead of getting your child used to one place and then when probation is done; okay now you have to go see someone else. I don’t think that helps when they have to talk to multiple people. I think that consistency and being familiar with the people you are going to talk to on a regular basis is very very important when it comes to mental health.” *Parent* “The most frustrating part was the really high turnover of probation officers, that it was difficult to get assistance…normally the child, they need someone that they can rely on consistently… there’s no consistency and how can you rely on someone who keeps changing? So if you build trust in one person, if they keep changing, how do you keep relying on the system that’s not being consistent with you?… Everything keeps changing, the only person that they learn to rely on is themselves, so then they start dismissing these outside factors, since they’re finding that the only consistent thing in their life is themselves, so then they keep, they just bottle everything up and they think I guess I have to handle everything myself. I think that’s how it kind of worked in her mind, is that she’s really self-reliant.”

Youth perceived barriers included lack of interest in treatment, concerns that treatment would not help them, negative experiences within the JJ system, lack of time for treatment, and trust issues with the system due to staff turnover or not knowing staff. Guardian perceived barriers included cost and insurance involvement, lack of communication for youth appointments and expectations, difficulty in finding appropriate treatment for the youth, and youth interest in treatment.

### Barriers perceived by youth

#### Interest in treatment

Many youths voiced that substance use treatment was not necessary. They either denied using substances or believed that their use did not constitute a problem. Some even mentioned that their drug of choice (most often marijuana) should not be prosecuted in the same way as other substances.

*“I just thought it was stupid. Other kids that do cocaine, heroin, speed, meth, and all that stuff. I’m just here smoking Mary Jane, ain’t nothing wrong with that. It’s legal in [some] states. Why can’t it be legal everywhere?”*

Some youths expressed concerns that treatment would not help them because it did not fit their learning style, or they did not want to attend. They discussed that real life does not match the work that was given to them in treatment and were doubtful that the things they were taught would make a difference in their lives.

*“No, not really [interested in treatment], because no matter what anybody’s telling, I’m going to do what I want to do at the end of the day. My head will tell me about training technique, this is not going to help, this is what will help in real life. If I try to think about something, -read a book and color, but in real life, ain’t nobody likely to tell me, ‘No. Wrong move. Read a book.’ It was just her telling me to think like that and I can’t just think like that.”*

Most of the youths did not want to go to treatment and felt that the treatment would be less effective because it was forced.

#### Negative Juvenile Justice (JJ) experience

A majority of youth were concerned about the impact of JJ experience on their mental health and substance use. The stress of the situation led some to desire to use, even if they did not otherwise want to.

*“But if anything, it [JJ experience] did push me to do it [use substances] more because of the stress of being arrested and the stress of all of these new issues that I’m facing and the worry of drug tests and everything else. It was a lot of stress on me at once, and I’m going to be bluntly honest with you; the day after I got arrested, I was like I’m just having consistent panic attacks. I’m like I need to smoke.”*

Unlike many guardians who expressed hope that this experience would give the youth pause from engaging in behaviors that would lead to recidivism, this was not the overall sentiment expressed by youth. They instead focused on effects of the JJ system on their past and current mental health and subsequent desire for use of substances.

Some youths also expressed concerns about the point of JJ services. The youth that discussed this point were unsure if the system was designed to help them with their healing process.

*“They [the system] don’t help you at all. They basically just want you put in the system. They don’t really try to help you as much as you think they’re going to help.”*

#### Scheduling/time

Many youths in this sample were employed at least part-time, which made scheduling required activities such as drug screening or treatment difficult.

*“I don’t go there [treatment] because I feel like I don’t have time. I have so much to do. I have a job. I go to school. I have all my friends. I don’t see how I would get that in there for real.”*

As stated in the quote above, many of these youth are expected to continue their normal activities with family, friends, school, and work, on top of meeting probation requirements. This reportedly added to their stress and reduced the likelihood that some of them would show up or complete treatment.

#### Trust

Some youth also struggled with opening up and felt that if they were unable to talk about their problems then the treatment would not work for them.

*“I don’t talk about the things that I need to talk about, because that’s just not something I share with anybody. I share that with people I trust, and I don’t trust very easily.”*

Additionally, many expressed dismay that they were shuffled between probation officers or that their therapist would leave due to turnover issues at both agencies. This added to the youths’ wariness with trust. This barrier is discussed more in the section on suggestions for improvement.

### Barriers perceived by guardians

#### Cost and insurance

One of the top themes within the guardian group was the cost of having a child placed in the JJ system and outside treatment. Many found treatment options that they felt would work for their child but that were unaffordable or not covered by insurance. One parent, in speaking about a rural residential treatment program stated:

*“It [treatment] was like just as expensive as sending him [youth] to college…Once they told me the price, I’m like, “Yeah, I cannot afford that. I couldn’t even afford that if I worked two jobs.”*

This particular guardian was discouraged because they felt that the program would have helped their child and engaged in a way that worked for their child, yet it was inaccessible. Other guardians felt that requiring the guardian to pay for drug screening and other probation requirements for their older youth (nearly age 18) was unfair and led to unreasonable cost.

#### Lack of communication from both JJ and Community Mental Health Center (CMHC)

Nearly all guardians discussed lack of communication as a large barrier to timely treatment. Some noted that the JJ system would communicate important information directly to the child instead of the guardian, leading to misunderstandings or a complete lack of transfer of information. Many in this group also mentioned missed therapy appointments or finding out about an appointment on the same day that they were to attend because of lack of communication.

*“I wake up in the morning, take him there [treatment facility] on Saturdays like I was instructed to. Then when I talked to the people there, nobody knew anything and no one had talked to anybody from juvenile probation. There was nothing in place.”*

Communication issues were the most mentioned frustration expressed by guardians. They felt left out of the process or in the dark about expectations.

*“And then they [CMHC] were supposed to get back with us and set up further appointments and they have not, and I’ve made calls to them and I’m just told that they will reach out to us.”*

#### No appropriate treatment available

Similar to the cost barrier, some guardians could not find treatment that was appropriate for their child. Available treatment may only provide services for substance use, or mental health treatment was not accessible at all. Many guardians mentioned long waiting lists and lack of communication if an appointment could not be scheduled at the time of arrest or release.

*“Every single facility that we called, there were waiting lists. And when we would tell them what we were needing to be seen for, they were very hesitant to schedule her and would say, “Well, we’ll put you on the list and we’ll call you back.””*

A few guardians discussed barriers from an overloaded system. They expressed that more severe cases were seen first, leaving their child without treatment.

*“When we would tell them [treatment facility] what she was she was needing it for, they’re like, “Oh, well, we actually do more of like an opiate, or if she had heroin abuse issues.” They were very booked on having to help people with more severe issues.”*

#### Youth interest

Like some of the youth interviews, guardians expressed that some youth were not interested in pursuing treatment. This made adding non-required treatment particularly challenging.

*“He [youth] said he really didn’t like it…he felt like it was just nonsense, like it was just a bunch of talking, and it didn’t help anything.”*

### Suggestions for improvement

#### Emphasis on mental health

Many guardians felt that the JJ system did not see mental illness playing a role in their child’s actions. They felt that the system should integrate mental health screenings and services into requirements for parole.

*“[JJ] should have some kind of program to where they help these children learn about their diagnoses and learn how to function with it. If they have real kids that are coming through the juvenile system with legit diagnoses. How come they don’t have a program that’s just like, listen, yes, you have extra challenges, but you need to learn how to live with them, how to cope with them. How to deal with them because once you enter society if you allow your diagnoses to take over your actions and behaviors, you are facing real consequences. Why don’t they have something like that?”*

Some guardians even felt that mental health issues were the sole reason of their child’s reason for arrest, and they were hopeful that with mental health improvements, their child would have a better outcome.

*“I said, that’s just the plain truth and the reality of it. Why are we saying, oh, remember, he has real diagnosis, because when he turns 18, those diagnoses don’t mean shit. All you have left to offer these people that have these mental behavioral challenges is jail and prison? This is what we’re doing with these people. We’re wasting tax dollars when they should definitely be some kind of coaching, teaching them.”*

#### Timely consequences

Some guardians felt that the JJ system did not give enough immediate consequences for their child’s actions. They suggested that having real-time consequences may prevent repeat offenses, and that allowing the youth to continue with certain behaviors prevented them from learning a lesson.

*“I [parent] actually think that they [JJ] were maybe too lenient on her, if she [youth] was to call every day for a drug test. That’s one of the things that she was supposed to do and then I would get the phone call saying that she needs to call in every day… there were some times that she did have her phone and he [probation officer] still contacted me, to have her keep on calling and she missed several days and really nothing was done.”*

#### Better communication

As mentioned under guardian barriers above, most felt that improved communication between the JJ system or CMHC would make the process easier and less stressful. It would also increase their chances of being able to drive the youth to their appointments without having to miss work at the last minute.

*“I guess just better communication [would make it easier to participate in treatment]. You know what I mean? It’s like when he did the assessment, I don’t know what was going to be recommended or anything like that. So, it’s like, I’m at a stand still trying to figure out what’s going on. What are they doing? You know what I mean?”*

#### Reduced financial burden

Like the guardian cost theme mentioned above, many guardians felt that the JJ system overcharged for required services. Some had youth who were nearly adults, and they felt that paying for their youth’s actions was not fair. Others felt that the services given were not worth the cost that was paid.

*“At the end of it, I [parent] had to pay [the county] money but I just felt like I shouldn’t have paid for anything because truthfully, they didn’t do much of anything… It was like the administrative fee. I don’t know maybe to type in what happened. I had to pay somebody to do that…I had to pay the court fees, but we never actually went to court.”*

#### Continuity of care

Per reports from parents and youth, most participants were on probation for only 3–6 months. With a lengthy waiting period for service initiation and discontinuation of services upon probation completion, several guardians reported that their youth was only able to receive services for a short period of time. Guardians felt that services should extend past the time of discharge from the JJ system to promote continuity of care, prevent recidivism, and build trust between the youth and their therapist.

*“So, I feel like if they [courts] can offer them service during probation they should be able to offer them past that instead of getting your child used to one place and then when probation is done; okay now you have to go see someone else. I don’t think that helps when they have to talk to multiple people. I think that consistency and being familiar with the people you are going to talk to on a regular basis is very, very important when it comes to mental health.”*

Another aspect of continuity of care involves staff turnover. Many guardians and youth expressed how challenging it is to develop trust in a probation officer or therapist, only to be assigned a new one after a few months and have to start the process all over again. One parent stated:

*“The most frustrating part was the really high turnover of probation officers, that it was difficult to get assistance…normally the child, they need someone that they can rely on consistently… there’s no consistency and how can you rely on someone who keeps changing? So, if you build trust in one person, if they keep changing, how do you keep relying on the system that’s not being consistent with you?... Everything keeps changing, the only person that they learn to rely on is themselves, so then they start dismissing these outside factors, since they’re finding that the only consistent thing in their life is themselves, so then they keep, they just bottle everything up and they think I guess I have to handle everything myself. I think that’s how it kind of worked in her [youth’s] mind, is that she’s really self-reliant.”*

#### Positive reinforcement

Many youth felt that their positive accomplishments while on probation were not recognized. They believed that periodic recognition of the positive steps they were taking would motivate them to continue down that path. As one youth said:

*“I feel like that’s a big step for me to stop and pass those drug tests, because I did it just because I didn’t want to be on probation or deal with them no more. But I feel like maybe if they would have, if they [JJ] give kids a little more recognition than they do I feel like some things wouldn’t go as bad as they do. Like, I know I shouldn’t want a reward for doing the right thing, but at the same time, sometimes people need that little extra push just to feel better about themselves and it would make them be like, okay, well they do recognize what I’m doing, maybe I should keep it up.”*

## Discussion

**Fig. 1:**
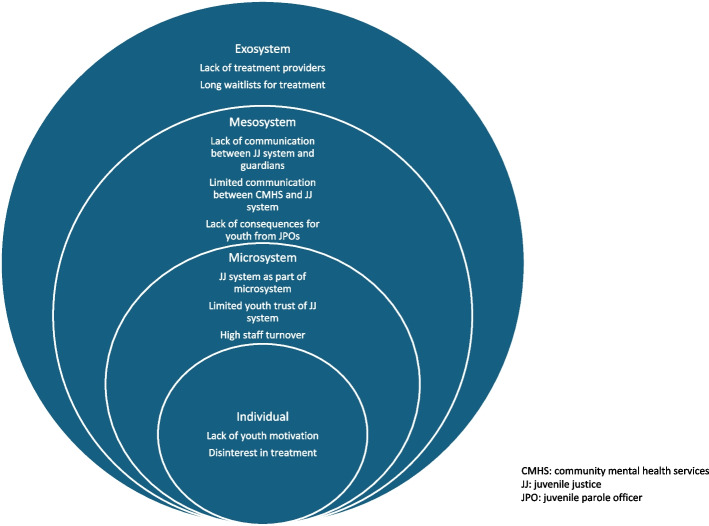
The ecological systems theory model

This study explores the barriers to substance use treatment that are experienced by JIY and their guardians and suggestions for improvement. Barriers were identified across multiple levels of the youths’ environment. In accordance with the ecological systems theory, many of these barriers appear to arise from interactions between the youth and their environment or between systems within their environment and the mutual influence that they have on each other (Fig. 1).

### Intrinsic youth factors

The largest barrier mentioned by youth was their own disinterest in treatment. The youth that did acknowledge a problem indicated they could solve the problem on their own so treatment was unnecessary. These findings are similar to previous studies that found challenges with symptom recognition and inflated self-reliance to be major barriers to youth seeking mental health treatment (Elkington et al., 2020; Gulliver et al., 2010). Parents also reported youth disinterest as a barrier, noting that if their youth did not want to participate in treatment, they could not force them to do so. Motivation is important to treatment success, but youth often have lower motivation for treatment than adults (Breda & Riemer, 2012), and the motivation that they do have is most commonly from external pressures (Goodman et al., 2011; Kerwin et al., 2015) which may be less effective than intrinsic motivation (Cornelius et al., 2017). Motivational interviewing is an effective technique that could be used by juvenile justice staff to help youth identify a need and encourage them to participate in treatment, although extensive training is needed to implement it successfully (Breda & Riemer, 2012). This may be especially useful if done prior to treatment initiation and continued throughout the first few weeks of treatment as youth motivation for treatment has been found to increase dramatically after these first few sessions (Merrill et al., 2017). Contingency management is another evidence-based treatment modality that can increase motivation in youth with SUD and can increase participation in other therapeutic treatments (Stanger & Budney, 2019).

### Microsystem factors

JIY have a unique environmental composition in which entities such as health and legal services that would typically exist in a child’s exosystem become a part of their daily lives. Interactions between the child and members of their microsystem are delicate and can promote positive outcomes or quickly become barriers. Distrust of the JJ and CMHC system was a common theme throughout this study. Trust is a vital component of all relationships and has historically been low between youth and the justice system (Elkington et al., 2020; Flexon et al., 2009). Negative JJ experiences, feeling let down by the system, and difficulties trusting new staff were common amongst youth. Youth requested positive reinforcement to mitigate issues of trust. Parents suggested decreased staff turnover. It is evident that trust is an important factor to treatment engagement and should be explored further.

### Mesosystem factors

Mesosystem barriers included all those that arise from interactions between a youth’s microsystems. A major concern that was brought up by parents was communication between JJ and CMHC staff for scheduling and treatment. In this study treatment services were provided to JIY through associated CMHCs rather than directly through the JJ system as is the case for most JJ centers (Funk et al., 2020; Saunders et al., 2016; Scott et al., 2019). Challenges in collaboration between these two systems is common. Strategies to mitigate these challenges or to provide more services in-house should be explored further.

Other concerns that were brought up by parents were Juvenile Probation Officers (JPOs) not providing enough timely consequences for youth, communicating through youth rather than to the parents directly, and being expected to pay fines that they felt the responsibility of should fall on the youth. This tension may be due to the overlap of microsystems caused by JJ intervention. During community supervision, JPOs and the JJ system itself take on a parenting role for youth (Feld, 2017; Samantha et al., 2018). This overlap of microsystems can create tension and change family dynamics due to JJ staff and parents having differing expectations of each other (Fine et al., 2020; Paik, 2016; Phelps, 2020). Efforts to create a collaborative relationship between JPOs and parents with clear expectations of each other may help to reduce some of these concerns.

### Exosystem factors

The most mentioned structural barrier was the lack of available treatment providers. Many JIY interviewed were unable to access treatment due to long waiting lists or prioritization of more severe cases. This is not unique to the individuals in this study. A recent study found that only one-third of JJ systems provide SUD programming (Funk et al., 2020) including both in-house programming and through affiliated CMHCs. Continuity of care and cost of care were concerns for parents as many services were no longer covered once a youth completed probation. These concerns are common to previous studies (Elkington et al., 2020; Iskra et al., 2018) and indicate a need for systemic change to reduce barriers to treatment access in this population.

Within the JJ system, it appears important to both youth and their guardians to have a seamless experience guided by consistency in expectations and accurate communication. Policy surrounding communication systems and consistent messaging to guardians would likely clear up major concerns expressed by those in this study. Working closely with CMHCs to provide care quickly while the youth is cared for by the JJ system (such as the care received during the parent implementation study, see Aalsma et al., 2019) could quiet guardian anxiety and allow for longer treatment during JJ involvement. This reduction in perceived barriers will lead to greater access to needed care.

### Limitations

There are several limitations to this study. Interviewers did not know what substance(s) were used by youth which made interviewing youth who claimed to never use substances challenging. Even though the youth were offered one of two programs depending on severity of substance use, this information was not readily available, so the interviewer relied on the memory of the youth or guardian about the program which was offered. Because the programs for this project were housed within a larger mental health system, some youth likely participated in mental health services without accessing the University-sponsored programming. This study involved primarily females, which is not representative of JJ involved youth as a whole. It is unclear why females were more likely to agree to study participation. Inter-coder agreement was not calculated for this study. This study is likely not generalizable to an international justice system, and even within the United States may not generalize outside of a rural or suburban justice setting. Finally, while every effort was made to standardize the content of the interviews, there were different levels of experience between researchers conducting the interviews.

## Conclusion

In conclusion, many barriers to substance use treatment exist, particularly with JIY who are at high risk of recidivism. Some of these barriers include cost, communication difficulties between youth, guardians, and the care system, child interest, trust issues among both youth and guardians with JJ and CMHCs, and lack of appropriate treatment availability. We suggest that increased communication and bridging youth to outpatient care during and after probation may decrease some of these barriers. It is important that we continue to work toward barrier elimination for this vulnerable population, so that youth can avoid recidivism and are able to and improve overall health and well-being.

## Data Availability

N/A.

## Abbreviations

SUD: Substance Use Disorder
JIY: Justice-involved youth
JJ: Juvenile justice
CMHC: Community mental health center
JPOs: Juvenile probation officers
